# Improving Health Equity for Spanish-Speaking Latine Communities: Community Priorities, Challenges, and Recommendations

**DOI:** 10.3390/ijerph23040472

**Published:** 2026-04-09

**Authors:** Sandy K. Aguilar-Palma, Lilli Mann-Jackson, Jorge Alonzo, Amanda E. Tanner, Thomas P. McCoy, Alain G. Bertoni, Omar Valera, Scott D. Rhodes

**Affiliations:** 1 Department of Social Sciences and Health Policy, Wake Forest University School of Medicine, Winston-Salem, NC 27157-1063, USA; lillimarie.mannjackson@advocatehealth.org (L.M.-J.); jorge.alonzo@advocatehealth.org (J.A.); scott.rhodes@wfusm.edu (S.D.R.); 2 Department of Public Health Education, University of North Carolina Greensboro, Greensboro, NC 27402-6170, USA; aetanner@uncg.edu; 3 Capstone College of Nursing, The University of Alabama, Tuscaloosa, AL 35487, USA; tpmccoy@ua.edu; 4 Division of Public Health Sciences, Wake Forest University School of Medicine, Winston-Salem, NC 27157-1063, USA; alain.bertoni@advocatehealth.org; 5 Public Health, Mecklenburg County Government, Charlotte, NC 28202, USA; omar.valera@mecklenburgcountync.gov

**Keywords:** Latine health, Spanish-speaking communities, community-based participatory research, health equity, COVID-19, community forum, culturally responsive care, social determinants of health

## Abstract

**Highlights:**

**Public health relevance—How does this work relate to a public health issue?**

- This study addresses persistent health inequities affecting Spanish-speaking Latine communities, including barriers to preventive care, mental health services, HIV prevention and treatment, disability services, and broader social determinants of health.
- Using a community-based participatory research and empowerment framework, this study examines how the COVID-19 pandemic exposed and intensified structural barriers such as language exclusion, immigration-related fear, medical mistrust, and limited access to culturally responsive services.

**Public health significance—Why is this work of significance to public health?**

- This study identifies eight community-defined priorities that provide an empirically grounded agenda for advancing health equity among Spanish-speaking Latine communities beyond COVID-19.
- Findings show that health inequities are shaped by interconnected structural, political, and institutional factors, underscoring the importance of community-driven approaches to understanding and addressing public health challenges.

**Public health implications—What are the key implications or messages for practitioners, policy makers and/or researchers in public health?**

- Public health practice should invest in bilingual and culturally responsive services, strengthen community health worker and community-based peer navigation models and improve cross-sector coordination to reduce barriers to care, build trust, and improve health outcomes.
- Policy and research efforts should address upstream determinants of health, including immigration-related fears, housing, food insecurity, employment, transportation, and political exclusion, through sustained community-engaged and structurally informed strategies.

**Abstract:**

Our community-based participatory research (CBPR) partnership convened an in-person, bilingual empowerment theory-based community forum to disseminate and translate findings from our trial of *Nuestra Comunidad Saludable* (Our Healthy Community), a multilevel intervention designed to improve uptake of COVID-19 testing and vaccination among Spanish-speaking Latine communities in North Carolina. The forum brought together community members, healthcare providers, organizational representatives, and academic researchers from across North Carolina. Drawing on findings from the intervention trial, participants engaged in facilitated, structured dialogue to identify community priorities and generate recommendations to advance health equity among Latine communities. Thirty-six participants identified eight priorities: (1) reducing health service gaps and inequities exposed by COVID-19; (2) expanding access to bilingual, culturally responsive mental health services; (3) improving understanding of HIV prevention and treatment; (4) strengthening services for children with disabilities; (5) protecting immigrant rights and ensuring safe access to services; (6) increasing political and social support for Latine health; (7) improving access to trusted, culturally responsive providers and community organizations; and (8) addressing social determinants of health, including employment, housing, and food security. The empowerment-based forum identified community priorities, challenges, and recommendations that can inform practice, intervention, policy, and research, and advance health equity for Spanish-speaking Latine communities.

## 1. Introduction

In the United States, Hispanic/Latine populations represent the second-largest demographic group after non-Hispanic Whites [1,2], yet face significant challenges to healthcare access [3,4,5]. These challenges were intensified during the COVID-19 pandemic [6,7,8]; between 2019 and 2021, Latine populations experienced a 5.1% decline in life expectancy, equivalent to a loss of 4.2 years, one of the steepest decreases among US demographic groups [9]. Key contributors to these inequities include language barriers, racial and ethnic discrimination, anti-immigrant sentiment and rhetoric, and restrictive immigration policies that discourage or limit service use [10,11,12,13].

These inequities are particularly salient in the US South, including North Carolina, which provides an important context for advancing health equity among Latine populations. Located in the southeastern United States, the state encompasses a mix of urban, suburban, and rural counties. In 2020, approximately 65% of North Carolinians lived in urban or regional center/suburban counties, while 35% lived in rural counties. Hispanic/Latine persons comprised over 12.0% of the state population in 2024, and the Latine population is geographically dispersed [14,15,16].

To address the unique needs and experiences of Latine communities during the COVID-19 pandemic, our community-based participatory research (CBPR) partnership developed, operationalized, implemented, and evaluated *Nuestra Comunidad Saludable* (Our Healthy Community), a Spanish-language intervention designed to increase COVID-19 testing and vaccination among Spanish-speaking Latine communities [17]. The intervention combined peer navigation with mobile health (mHealth) strategies, leveraging in-person helping, social media, and text messaging to disseminate information, offer support, and reduce barriers related to COVID-19 testing and vaccination. Twenty Spanish-speaking community leaders were recruited to serve as peer navigators, known as *Navegantes*; each engaged eight members of their social networks, for a total of 160 participants. *Navegantes* coupled with their social networks were randomly assigned to either the intervention or delayed-intervention group. Intervention-group *Navegantes* were then trained to conduct formal and informal outreach activities for six months, closely collaborating with the research team for ongoing feedback, problem solving, activity planning, and strategy refinement. Data were collected from social network members at baseline and 6-month post-intervention follow-up, with a retention rate of 98.1% at follow-up. At follow-up, intervention-group participants reported significant improvements in several intervention-related psychosocial determinants, including testing intention and testing barriers [18,19].

The intervention yielded significant improvements in key psychosocial and access-related outcomes, including increased intention to test for COVID-19 and reduced perceived testing barriers. Participants in the intervention group had higher odds of intending to test for COVID-19, lower odds of reporting any testing barriers, and fewer perceived testing barriers overall. Although not statistically significant, both study groups showed meaningful increases in being up to date on recommended vaccine doses six-month follow-up. The intervention did not significantly affect COVID-19 testing uptake or vaccination uptake at six-month follow-up. Process evaluation findings indicated high exposure to Navegante-delivered education, although direct assistance with linkage to testing, vaccination, and treatment services was less consistently reported [19].

Taken together, these findings demonstrate the intervention’s impact on key antecedents to behavior change while highlighting opportunities to strengthen linkage to services. The current study builds on the *Nuestra Comunidad Saludable* intervention by examining an in-person, bilingual, empowerment theory-based community forum convened to disseminate findings and support collective reflection and action. After the intervention trial concluded, we organized an empowerment theory-based community forum [20] to synthesize lessons from the COVID-19 pandemic and the implementation of the intervention, and to identify community priorities, challenges, and recommendations to advance health equity among Spanish-speaking Latine communities. Although our partnership has previously used empowerment theory-based community forums as a CBPR dissemination and translation strategy, the current study is distinct in applying this approach to findings from an intervention trial and in using the forum to identify broader, community-defined priorities for advancing health equity among Spanish-speaking Latine communities beyond COVID-19.

## 2. Materials and Methods

Our CBPR partnership convened an in-person, bilingual empowerment theory-based community forum [20] to disseminate and translate findings from our trial of *Nuestra Comunidad Saludable*. The community forum convened members of our CBPR partnership and the *Navegantes* from the *Nuestra Comunidad Saludable* intervention and a diverse group of stakeholders, including community members, healthcare providers, representatives from health departments and other community organizations, and academic researchers. The invited stakeholders were purposively selected by the CBPR partnership based on their experience supporting Spanish-speaking communities in North Carolina, United States, particularly through COVID-19 response efforts and related health and social service programs. Potential participants were identified through existing partnership networks and prior collaboration with community-based organizations, public health agencies, and service providers involved in outreach, testing, vaccination, and broader community support during the pandemic. Recruitment aimed to include individuals representing diverse sectors and perspectives relevant to Spanish-speaking Latine communities. Stakeholders were invited by email by members of the research team and community partnership, with follow-up communication as needed to confirm participation. We did not systematically document refusals or reasons for non-participation.

The foundation of the community forum process is empowerment education theory which emphasizes that meaningful change requires both learning and critical reflection to foster awareness and promote informed action [20,21]. Our partnership developed, refined, and successfully applied this method to disseminate and translate findings into action in several previous studies, including: (1) a qualitative study on sexual risk among racially and ethnically diverse gay, bisexual, queer, and other men who have sex with men (GBQMSM) [22]; (2) a mixed-methods study on the impact of immigration enforcement on Latine health [23]; (3) a qualitative study to explore barriers to healthcare utilization and identify potentially effective intervention strategies to increase health and well-being among members of Korean American communities [24]; and (4) a study to develop and evaluate an intervention to reduce HIV and sexually transmitted infection disparities and address social determinants of health among young GBQMSM and transgender women of color [25].

The community forum was held in person on a weekday in September 2024, at the Mecklenburg County Health Department in Charlotte, North Carolina, United States, a familiar and accessible site for participants. To ensure full linguistic accessibility and participation, professional simultaneous Spanish-English interpretation was provided throughout the event. Given the sensitive nature of immigration-related concerns discussed during the forum, the forum was conducted in a linguistically accessible format with bilingual support, and data documentation relied on written notes rather than audio-recording to support participant comfort and reduce concerns about disclosure. Consistent with our community-engaged approach, these decisions were guided by the ethical need to balance rich data collection with the comfort and protection of participants and organizations in a context marked by mistrust and potential sociopolitical risk.

To support participation from across the state and minimize time burden, the forum was structured as a half-day event, consisting of a three-hour session followed by a networking lunch to further foster relationship-building among participants. The agenda featured an overview of intervention development and implementation, a summary of primary study findings, and a panel discussion in which *Navegantes* shared their experiences implementing the intervention. The session culminated in a facilitated discussion that engaged all participants in sharing perspectives on practice, intervention, policy, and research priorities to advance health equity among Spanish-speaking Latine communities.

Our partnership has developed a structured set of steps to facilitate inclusive, action-oriented dialogue during community forums [16,17,19,20]. These include sequential prompts that move from (1) concrete reflection to (2) critical interpretation and ultimately to (3) action-oriented analysis. The discussion portion of the forum opened with concrete questions, such as “What reactions do you have to the information shared about this project?”, “What stands out to you or feels familiar?”, “What parts of the findings seem unclear or surprising?”, and “In what ways does what you heard make sense or not make sense to you?” to help participants process the information presented, make connections to lived experience, and surface initial insights. The facilitator then shifted toward more interpretive questions, including “Why do you think these challenges exist?”, “Who is most affected, and why?”, and “What factors shape these issues, and how?” Finally, the discussion moved to explicitly action-oriented questions such as “What are the main health needs and priorities of Latines in North Carolina?” and “How could experiences with the *Nuestra Comunidad Saludable* intervention be applied to other health needs and priorities within Latine communities in North Carolina?”, as well as “What actions could community organizations, clinics, or health departments take to address these needs and priorities?” These latter questions encouraged participants to move from reflection to generating strategies to act upon findings.

During the discussion, one member of the team recorded key points on easel pads and kept them visible to all participants. This allowed participants to review, clarify, and confirm these points in real time, providing immediate revision and validation of the information captured. In addition, members of our team took detailed notes during the forum to capture participant comments and feedback. After the forum, two team members (SDR and SKAP) reviewed and compiled the easel-pad content and note-taker notes into a single analytic document. Using constant comparison, they examined the data iteratively, grouped related ideas, identified recurring patterns, and developed thematic categories [26,27,28]. These preliminary themes were then reviewed and discussed with the broader research team to refine category boundaries, challenge interpretations, and ensure alignment between the recorded discussion and final themes. Ideas generated through this process were synthesized to identify community priorities, challenges, and recommendations to advance health equity among Spanish-speaking Latines. Analytic rigor was strengthened through team-based interpretation and through real-time participant validation during the forum, as the key points were visibly documented and could be reviewed, clarified, and confirmed by participants as the discussion unfolded.

The forum was organized and results analyzed by members of a multidisciplinary CBPR partnership with extensive experience working with Spanish-speaking Latine communities in North Carolina. Several team members had longstanding relationships with community partners and some forum participants through prior collaborative research and community engagement, including the *Nuestra Comunidad Saludable* intervention. We recognize that these relationships likely shaped both data generation and interpretation by facilitating trust and openness, while also creating the need for reflexive attention to assumptions. To address this, multiple team members participated in note-taking, synthesis, and interpretation, and emerging themes were reviewed and discussed by the broader team to refine analysis and ensure that interpretations remained grounded in the forum discussion.

### Ethics

The Wake Forest University School of Medicine Institutional Review Board approved all study protocols for the *Nuestra Comunidad Saludable* intervention trial. Ethical approval code: IRB00079510; original approval date: 2 May 2022.

## 3. Results

### 3.1. Participants

The community forum included 36 participants, representing a diverse range of perspectives and experiences. Participants included community organization representatives (*n* = 12), *Nuestra Comunidad Saludable Navegantes* (*n* = 10), academic institution representatives (*n* = 9), county health department staff (*n* = 4), and a local Spanish-language media representative (*n* = 1).

### 3.2. Community Priorities, Challenges, and Recommendations

Forum discussions generated eight community priorities, each accompanied by specific challenges and actionable recommendations for addressing health equity among Spanish-speaking Latine communities in North Carolina (Table 1). Table 1 provides a summary of the community priorities, challenges, and recommendations identified, while the narrative below offers more detailed explanation and context for each priority area. Each final priority area represents a synthesis of related concerns, examples, and action ideas raised across the forum discussion and grouped through an iterative team-based thematic analysis of easel-pad notes and detailed written notes.

#### 3.2.1. Reducing Health Service Gaps and Inequities Exacerbated by the COVID-19 Pandemic

Participants described pervasive and multilevel gaps in health services that were both exposed and exacerbated by the COVID-19 pandemic. These gaps included limited awareness of prevention services, wide-spread misinformation disseminated through religious organizations and media and by word-of-mouth, originating in both the United States and countries of origin, and deepening mistrust of the US healthcare system. Participants emphasized that these challenges were compounded by structural barriers such as lack of health insurance, particularly for those with undocumented immigration status, limited understanding of how to use available coverage, and difficulties navigating complex and fragmented and complex healthcare systems. Chronic shortages of bilingual and culturally responsive providers with further limited access to accurate information and to care.

These barriers were described as particularly pronounced in rural and geographically isolated communities where many Latines live and where access to services, community organizations, and trusted sources of information is limited. Participants also identified COVID-19 fatigue and political polarization as cross-cutting factors that undermined prevention efforts. Participants highlighted ongoing needs for access to testing, guidance from trusted experts, and preparation for future public health emergencies. Across themes, participants highlighted that challenges were often amplified in rural and geographically isolated communities, where fewer services, trusted organizations, bilingual providers, transportation options, and other supports were available.

To address these multilevel barriers, participants recommended strengthening and sustaining community health worker programs, including providing appropriate compensation, integrating them across health systems, and aligning them with care teams. Participants emphasized the importance of improving health literacy through trusted outreach such as door-to-door engagement and face-to-face education as well as the need for simple, clear health messaging delivered in Spanish and other languages spoken within Latine communities, including Indigenous languages (e.g., Mayan languages).

Participants also highlighted the importance of leveraging accessible communication platforms (e.g., text messaging, WhatsApp, video sharing, and Facebook Live) to facilitate timely and wide-spread dissemination of information. Additional recommended strategies included expanding bilingual and bicultural services, strengthening language access infrastructure, and increasing workforce diversity; building cross-sector partnerships and coalitions to coordinate health efforts across organizations and regions; and ensuring that rural Latine communities are intentionally included in home visits, outreach, services, and emergency preparedness planning.

#### 3.2.2. Expanding Access to Bilingual, Culturally Responsive Mental Health Services

Participants identified substantial unmet mental health needs within Spanish-speaking Latine communities, describing long-standing gaps in awareness of mental health conditions and available services. They emphasized that immigration-related stresses, including fear of deportation, concerns about disclosing immigration status, uncertainty about eligibility for services, and worries related to family separation, create significant psychological burden and often prevent members of Latine communities from seeking help. Participants noted that stigma surrounding mental health, reinforced by both cultural norms and misinformation, including from religious institutions, further discourages open discussion and utilization of services.

Structural barriers were described as major contributors to unmet needs, including limited availability of bilingual and culturally responsive mental health providers resulting in long wait times for appointments, lack of insurance or inability to afford care, and the absence of accurate and linguistically appropriate information. Participants noted that mental health challenges intensified during the COVID-19 pandemic as isolation, job loss, and chronic stress amplified anxiety, depression, and family strain while access to in-person support became more limited.

To address these barriers, participants recommended normalizing mental health care through clear, culturally responsive education campaigns delivered by trusted community organizations and faith-based groups. They emphasized the importance of disseminating information about free or low-cost mental health services in Spanish and other languages. Participants recommended expanding the workforce of bilingual and culturally responsive mental health providers, strengthening community health worker roles to include mental health outreach, and incorporating mental health support into community-based programs such as through collaborations with faith-based institutions and faith leaders. Additional strategies included using texting, video sharing, and other digital tools to reach isolated community members; supporting bilingual Latines in helping monolingual Spanish speakers and those who speak Indigenous languages learn about and navigate services; and increasing partnerships with clinics, schools, and community organizations to improve early identification of mental health needs and streamline pathways to care.

#### 3.2.3. Improving Understanding of HIV Prevention and Treatment

Participants described substantial gaps in knowledge and understanding of HIV prevention, testing, and treatment within Spanish-speaking Latine communities. They noted that limited awareness of evidence-based prevention options, particularly pre-exposure prophylaxis (PrEP), and misconceptions about HIV transmission continue to fuel stigma and discourage Latines from seeking testing or care. Participants emphasized that stigma is exacerbated by misinformation, including religious and culturally rooted narratives that frame HIV as taboo or morally charged, making open discussion difficult for many community members.

Structural and system-level barriers were also highlighted. Participants described how language constraints and the limited availability of bilingual, culturally responsive HIV providers reduce opportunities for timely testing, care linkage, and ongoing support for individuals living with HIV. Immigration-related fears, including concerns about disclosing personal information or interacting with government services, further deter individuals from accessing HIV-related services. Broader gaps in preventive care among Latine communities, including low familiarity with routine screenings, were also noted as contextual factors that contribute to underutilization of HIV prevention and treatment services.

To address these challenges, participants recommended increasing community-led HIV education delivered through trusted organizations, including faith-based groups, community centers, and Latine-serving nonprofits. They emphasized the need for clear, culturally responsive messaging to improve understanding of HIV prevention strategies, including PrEP, HIV testing, and treatment as prevention (i.e., Undetectable = Untransmittable). Participants also called for multilevel stigma-reduction efforts, such as community forums, peer-led discussions, and social media campaigns that normalize conversations about HIV and counter misinformation. Additional strategies included expanding bilingual HIV prevention services, strengthening care linkage pathways, incorporating Spanish-speaking community health workers and peer navigators into HIV outreach to foster trust and facilitate continuity of care, utilizing digital platforms to provide HIV-related information and support, and improving supportive services for people living with HIV, such as counseling and navigation assistance to promote treatment adherence and retention in care.

#### 3.2.4. Strengthening Services for Children with Disabilities

Participants described substantial challenges accessing appropriate services for children with disabilities and special health needs, noting that Spanish-speaking Latine families often struggle to locate, understand, and navigate the complex and fragmented systems responsible for supporting their children. Many families reported limited awareness of available services, across healthcare, education, and social service sectors, and emphasized that information is rarely provided in clear, linguistically appropriate formats. As a result, families frequently feel isolated, overwhelmed, and uncertain about how to advocate for their children’s needs.

Participants stressed that language barriers, a shortage of bilingual and culturally responsive providers, and insufficient outreach tailored to Latine communities further widen gaps in access. They noted that services for autism, developmental delays, and other disabilities are particularly difficult to obtain, with long waitlists and limited coordination between schools, clinics, and community programs. These structural challenges often force families to navigate multiple disconnected systems on their own, increasing stress and delaying essential supports for children and families.

To address these barriers, participants recommended improving dissemination of clear, accessible information about disability services in Spanish and other languages spoken within Latine communities. They emphasized the need to expand comprehensive, coordinated care models that streamline referrals and support families across health, educational, and social service settings. Participants also recommended increasing provider training to help clinicians and service providers better understand the cultural, linguistic, and lived experiences of Spanish- and Indigenous language-speaking Latine families. Strengthening partnerships among healthcare systems, schools, early intervention programs, community organizations, and community health workers were identified as critical for ensuring timely, continuous, and family-centered care for children with disabilities.

#### 3.2.5. Protecting Immigrant Rights to Ensure Safe Access to Services

Participants emphasized that immigration-related concerns profoundly shape whether and how Spanish-speaking Latine communities engage with health and social services. They described a pervasive climate of fear, driven by concerns about deportation and family separation, uncertainty about confidentiality protections, and anxiety about disclosing personal or household information, that leads many individuals, including those in mixed immigration status families, to delay or avoid care altogether. Participants noted that these worries extend beyond medical services to public programs, mental health care, and social supports, contributing to worsening health conditions, chronic stress, and widening inequities.

Participants described the political environment as an ongoing source of instability and uncertainty, citing specific policies such as North Carolina House Bill 10 (HB 10) which became law on 1 December 2024, and requires sheriffs to comply with ICE detention requests for undocumented individuals in their custody, as well as shifting federal and state immigration priorities. Participants emphasized that election cycles, media narratives, and policy debates intensify fear in immigrant communities, further reducing trust in institutions and discouraging service use. Participants also noted that misinformation about immigration and eligibility for services circulates widely, creating additional confusion and barriers to care.

To address these challenges, participants recommended actively promoting trusted, Spanish-speaking providers through community organizations and Spanish-language media. They emphasized the importance of offering clear, culturally responsive education in Spanish and other languages about confidentiality protections, patient rights, and available services that can be safely accessed regardless of immigration status. Strengthening partnerships between healthcare systems, community organizations, legal aid providers, and faith-based institutions was identified as critical for building trust and improving navigation support for both insured and uninsured individuals. Participants also called for coordinated community organizing and policy advocacy to safeguard immigrant rights, advance driver’s license access for immigrants, support mixed-status families, and ensure that health and social services remain accessible, welcoming, and safe for all.

#### 3.2.6. Increasing Political and Social Support for Latine Health

Participants emphasized that Spanish-speaking Latine communities often remain invisible within state and local health priorities, resulting in chronic underfunding, inconsistent attention, and limited policy action to address their needs. They described how this lack of sustained investment and meaningful representation in decision-making processes contributes to persistent health inequities and leaves many community priorities unaddressed. Participants noted that political and institutional dynamics, including competing health system interests and changing state and federal agendas, further complicate coordinated efforts to improve Latine health.

Participants highlighted how siloed initiatives across healthcare, public health, education, and community organizations weaken the collective influence needed to advocate effectively for Latine communities. Without coordinated advocacy, they explained, policies that disproportionately harm Latine immigrants, such as HB 10 and other immigration-enforcement efforts, are more likely to deepen mistrust and undermine access to services. They emphasized that political leadership at all levels, from federal to local, shapes the conditions under which Latine families seek care, work, attend school, and engage with public systems.

To strengthen political and social support for Latine health, participants recommended mobilizing community organizations to advocate collectively for equitable funding and policy change, including advancing culturally responsive and linguistically appropriate care, expanding access to services, and protecting mixed-status families. They emphasized the importance of building the capacity of Latine leaders, grassroots groups, and community members to participate in policymaking processes and to hold decision-makers accountable, including by sharing relevant research findings. Participants further recommended creating statewide partnerships and coalitions to share strategies, amplify community voices, and ensure that Latine health priorities remain visible and influential. Sustained, long-term advocacy was seen as essential to securing the investments and structural changes necessary to promote health equity for Latine communities across North Carolina.

#### 3.2.7. Improving Access to Trusted, Culturally Responsive Providers and Organizations

Participants described significant challenges navigating the fragmented and uncoordinated landscape of services available to Spanish-speaking Latine communities. Families must move across multiple disconnected systems (e.g., healthcare, social services, schools, and community programs) without clear referral pathways or communication among providers. This fragmentation leads to confusion, gaps in care, duplication of efforts, and missed opportunities for preventive services. Participants emphasized that these barriers are especially pronounced for newly arrived immigrants, families with limited English proficiency, and those living in rural or geographically isolated areas where fewer resources and trusted organizations exist.

Moreover, a persistent shortage of trusted, bilingual, and culturally responsive providers was highlighted as a central barrier to care. Participants noted that the availability of interpreters alone is insufficient; instead, Latine community members need providers and frontline staff who reflect their languages, cultural backgrounds, and lived experiences. Beyond direct care, participants emphasized a broader need for “language justice” ensuring that services, materials, and communication are accessible across the languages spoken by Latine communities, including Indigenous languages. Participants also raised concerns about the lack of a coordinated workforce pipeline to prepare more bilingual and bicultural Latine professionals across healthcare and social service roles.

To improve access, participants recommended strengthening partnerships and coalitions among healthcare systems, community organizations, faith-based groups, schools, and other entities trusted by Latine communities. Improved cross-sector communication and streamlined referral processes were viewed as essential to reducing fragmentation and ensuring families receive timely, continuous, and comprehensive support. Participants suggested developing community asset maps to identify bilingual providers, trusted organizations, community health workers, and other resources that can serve as anchors for Latine families. They further recommended expanding bilingual and culturally responsive services; improving recruitment, hiring, and retention of bilingual and bicultural providers and staff; creating pipelines and workshops to support Latine students entering health professions; and ensuring consistent, linguistically appropriate information and communication across all service systems.

#### 3.2.8. Addressing Social Determinants of Health

Participants emphasized that the social determinants of health, including employment opportunities, housing stability, food security, transportation, and overall economic conditions, play a vital role in shaping the health and well-being of Spanish-speaking Latine communities. They described how financial instability, unstable or overcrowded housing, and limited access to affordable, nutritious food create chronic stress and directly contribute to elevated rates of chronic diseases such as diabetes, obesity, and cardiovascular disease. Further, participants highlighted that many Latine families face competing demands, including long work hours, multiple jobs, transportation barriers, and childcare responsibilities, that limit their ability to seek preventive care, adhere to medical recommendations, or engage in physical activity and healthy eating.

These structural barriers were described as deeply intertwined with immigration-related stress, discrimination, limited employment protections, and underinvestment in communities where many Latine families live. Participants noted that rural communities often face even greater obstacles, including fewer grocery stores, limited public transportation, shortage of healthcare providers, and reduced availability of social services. They emphasized that addressing chronic disease prevention in Latine communities requires confronting these upstream social and economic conditions rather than relying solely on individual-level behavior change.

To advance meaningful progress, participants called for stronger cross-sector collaboration among public health leaders, healthcare systems, housing agencies, workforce development programs, food security organizations, and community-based groups. They recommended expanding affordable housing initiatives, increasing access to culturally grounded nutrition programs, supporting workforce training and pathways to living-wage jobs, and improving transportation and childcare supports. Participants also encouraged integrating social needs screening and referral processes into both clinical and community settings to better connect families with resources for food, housing, employment assistance, and financial support. Addressing these structural determinants, they noted, is critical not only for improving overall health but also for reducing the disproportionate burden of chronic disease experienced by Spanish-speaking Latine communities.

## 4. Discussion

Aligned with CBPR, the empowerment theory-based community forum created a space where Spanish-speaking Latine community members and partners could reflect on lived experiences, analyze the structural conditions affecting Latine health, and collaboratively identify actionable priorities, challenges, and recommendations. Consistent with empowerment theory [20,21,29], the forum emphasized that sharing information alone is insufficient; meaningful change requires participatory dialogue that validates community expertise, confronts social and structural barriers, and fosters collective action. Empowerment theory emphasizes that change is fostered not only through information-sharing, but through critical reflection, dialogue, recognition of lived expertise, and the collective development of action-oriented responses [29]. In this study, the forum reflected these core principles by creating a space in which participants moved beyond reacting to intervention findings to collectively analyzing the structural conditions shaping Latine health and identifying practical and policy-relevant strategies for change. These priorities represent a consensus-oriented snapshot of what participants viewed as most urgent and feasible in their local context. The recommendations that emerged, such as expanding and sustaining community health worker programs, strengthening culturally responsive and linguistically appropriate services, improving cross-sector coordination, and advancing multilevel advocacy, demonstrate the depth of community insight and the grounding of proposed solutions in real-world experience.

Findings from the forum underscore the severity and interconnectedness of the health challenges facing Spanish-speaking Latine communities. Participants described how long-standing inequities were exposed and intensified during the COVID-19 pandemic, including limited access to preventive services, partly due to challenges navigating complex health service systems that are often non-Spanish friendly, along with pervasive misinformation, deepening medical mistrust, and persistent shortages of culturally responsive providers. These inequities were particularly acute in rural and geographically isolated communities and among uninsured individuals, undocumented immigrants, and families with mixed immigration statuses, in which differential legal status within the household shapes access to services and exposure to policy-related stressors.

Specific health issues were highlighted as primary concerns. Mental health emerged as a major priority, with participants highlighting pervasive stigma, immigration-related stress, religious misinformation, and a shortage of accessible mental health services. Similarly, substantial gaps in understanding HIV prevention and treatment, driven by stigma, misinformation, and limited access to trusted providers, point to the continued need for culturally responsive, community-led HIV education and linkage-to-care strategies. Participants also identified groups whose needs are frequently overlooked, including children with disabilities, who experience fragmented services, inadequate information in linguistically appropriate formats, and barriers navigating systems not designed with cultural responsiveness in mind. While these findings are grounded in North Carolina, many of the barriers and priorities identified, including language exclusion, fragmented systems of care, immigration-related fear, workforce shortages, and unmet social needs, are likely relevant to Spanish-speaking Latine communities and other immigrant communities in many regions of the United States. More broadly, this study suggests that empowerment-oriented community forums may serve as a useful model for translating intervention findings into locally grounded but broadly informative public health action. Also, the forum did not explicitly compare rural and urban contexts as separate analytic categories, participants consistently emphasized that many of the barriers discussed were intensified in rural and geographically isolated communities, where services, trusted organizations, transportation, and culturally responsive providers were often more limited.

Although the forum occurred prior to the current escalation in immigration enforcement and shifting policies, immigration-related fears, ranging from concerns about deportation and family separation to mistrust of government systems and uncertainty about confidentiality, were described as powerful deterrents to accessing health, mental health, and social services. Since then, intensified anti-immigrant rhetoric and new federal and state actions have deepened fear and mistrust in Latine communities, making these findings even more relevant and underscoring the urgency of addressing structural and sociopolitical barriers to health and care. These fears intersect with broader social determinants such as food insecurity, unstable housing, precarious employment and low wages, and limited transportation, illustrating how structural vulnerabilities compound health inequities and contribute to chronic disease burden.

The political environment was recognized as a significant determinant of health and access to services. Participants noted that ongoing immigration-related policy enforcement and rhetoric contribute to heightened instability among Latine communities. The recent passage of HB 10 was cited as an example of how state-level policies amplify these concerns and further suppress service use. In this context, participants emphasized that political leadership at all levels has tangible consequences for the health, safety, and well-being of Latine families.

A consistent theme was the lack of political visibility and sustained investment in Latine health. Participants described how limited recognition of Latine health needs in policy agendas, insufficient representation in decision-making processes, and siloed organizational efforts collectively weaken efforts to achieve health equity. To counter these challenges, participants emphasized the importance of coordinated advocacy, coalition-building, capacity strengthening for Latine leaders, and sustained community organizing to elevate Latine health priorities and hold policymakers accountable.

Collectively, these findings highlight the need for multilevel, structurally informed approaches that extend beyond immediate healthcare needs. Recommendations emphasize upstream solutions: expanding culturally responsive and linguistically appropriate services; strengthening community health worker and community-based peer navigation models; improving public health communication and combating misinformation; enhancing cross-sector collaboration; protecting immigrant rights and normalizing non-English communication; and addressing foundational social determinants such as housing, employment, food security, and transportation. These insights align with a growing body of evidence demonstrating that equitable and effective public health efforts must meaningfully engage communities, confront structural racism and xenophobia, and respond to the broader political and social conditions shaping health.

Overall, the forum’s priorities and recommendations reflect the most pressing needs of Spanish-speaking Latine communities across North Carolina and offer a clear roadmap for practice, research, intervention development, and policy change. Future public health efforts should build on these insights to strengthen trust, expand access to culturally responsive and linguistically appropriate care, and advance health equity for Latine communities, particularly in a sociopolitical climate where immigrant families face renewed uncertainty, heightened vulnerability, and structural exclusion.

## 5. Limitations

This study has several limitations. The community forum included a small group that may overrepresent individuals already connected to services and advocacy, and discussions were shaped by the *Nuestra Comunidad Saludable* intervention trial focused on COVID-19 testing and vaccination, potentially prioritizing service-related concerns. Therefore, findings reflect the perspectives of forum participants rather than the full diversity of Spanish-speaking Latine communities in North Carolina. Because it was a one-time forum, priorities capture a single time point; future work could include a brief follow-up survey or a subsequent forum to assess the impact of the current US administration. As a group-based method, the forum may have been influenced by uneven participation, dominant voices, and social desirability, which may have shaped which priorities and recommendations were expressed most prominently.

## 6. Conclusions

This community forum provided a critical opportunity to center the voices of Spanish-speaking Latine community members and partners about the most urgent health needs and the structural conditions shaping the health of Spanish-speaking Latine communities in the wake of the COVID-19 pandemic. The priorities underscore the interconnected nature of healthcare access, mental health, HIV prevention and care, disability services, immigration-related concerns, and broader social determinants of health.

Rather than isolated service gaps, participants described a landscape shaped by structural inequities, political marginalization, and social exclusion. Their recommendations call for sustained investment in culturally responsive and linguistically appropriate services; expanding community health workers and peer navigation; coordinated cross-sector partnerships; and policies that protect immigrant communities and advance equitable funding and representation. Together, these findings reinforce that advancing health equity for Spanish-speaking Latine communities requires more than programmatic improvements; it calls for sustained structural, political, and institutional change led by and accountable to the communities most affected to restore trust and fix the service gaps caused by restrictive immigration policies.

## Figures and Tables

**Table 1:** Community priorities, challenges, and recommendations identified by forum participants.

Community Priorities	Challenges	Recommendations
1. Reducing health service gaps and inequities exacerbated by the COVID-19 pandemic	Limited awareness about COVID-19 and other health issues COVID-19 misinformation from both US and countries of origin Medical mistrust and mistrust of government institutions Difficulty navigating health insurance, including for individuals who have insurance but do not know how to use it Lack of insurance and services for undocumented community members (i.e., individuals without authorized immigration status) Limited bilingual, culturally responsive providers and staff across health systems Geographic barriers, especially in rural or remote areas where many Latines live COVID fatigue (e.g., “Not another vaccine”) Ongoing lack of preparedness for future emergent health issues	Strengthen community health work programs; ensure community health workers are paid and supported long-term Door-to-door and face-to-face outreach to improve health literacy Improve simple, clear health messaging using trusted messengers Continue access to COVID-19 tests and trusted experts Use texting and video sharing through WhatsApp, Facebook Live, and other social media for health communication Offer bilingual/bicultural services and multilingual materials (including Mayan and other Indigenous languages) Build partnerships and coalitions to coordinate work across organizations Conduct home visits for serious needs Expand outreach and service provision in rural/remote communities Include Latine communities in emergency preparedness planning
2. Expanding access to bilingual, culturally responsive mental health services	Limited awareness of mental health issues and available services Immigration-related stress, trauma, and fear Stigma related to discussing or seeking mental health care Shortage of bilingual/culturally responsive mental health providers Lack of insurance or inability to afford care Misinformation about mental health Isolation and disconnection from services	Normalize mental health care through education campaigns using trusted organizations Provide clear information about free/low-cost mental health services Increase bilingual and culturally responsive mental health providers Strengthen community health worker roles to include mental health outreach Include faith-based institutions and faith leaders in mental health education Use texting, phones, and video tools for mental health outreach Build capacity among English-speaking Latines to support monolingual Spanish speakers Increase partnerships among clinics, schools, and community organizations
3. Improving understanding of HIV prevention and treatment	Limited knowledge of HIV, PrEP, testing, and treatment Stigma and reluctance to discuss HIV openly Misinformation from religious or political sources Language barriers and insufficient bilingual HIV prevention providers Fear tied to immigration status Limited preventive care utilization among Latines	Provide community-led HIV education through trusted, culturally grounded organizations Increase PrEP and HIV treatment literacy Implement multilevel stigma-reduction initiatives Expand bilingual HIV prevention services Strengthen linkage-to-care, follow-up, and peer support services Use community health workers and digital platforms to promote accurate HIV information and support
4. Strengthening services for children with disabilities	Limited awareness of available disability and autism services Fragmented systems across medical, educational, and social services Difficulty navigating complex service pathways Feelings of isolation and lack of clarity among Latine families about how to advocate for their children Limited bilingual/culturally responsive outreach. Long waitlists for services	Improve dissemination of clear, culturally and linguistically appropriate information Expand coordinated care and streamline service pathways Train providers to support Spanish-speaking and Indigenous language-speaking Latine families Increase partnerships among schools, clinics, and community organizations Include disability support as part of community health worker and outreach programs
5. Protecting immigrant rights and ensuring safe access to services	Fear of deportation or immigration enforcement Concerns about confidentiality and disclosure of immigration status Avoidance of public programs due to fear Chronic stress related to immigration and family separation Additional uncertainty in election years (e.g., HB 10 and policy changes) Misinformation about immigration and eligibility for services	Identify and promote trusted, immigrant-friendly providers through community organizations Provide Spanish-language education about confidentiality and patient rights Strengthen partnerships with faith-based institutions, advocacy groups, and legal aid organizations Improve navigation support for both insured and uninsured individuals Engage in community organizing and collective advocacy to influence policy
6. Increasing political and social support for Latine health	Limited political visibility of Latine health issues Chronic underfunding of programs serving Latine communities Political and business tensions (e.g., between competing local health systems) affecting funding decisions Siloed organizational efforts limiting advocacy impact Leadership gaps and lack of community representation	Mobilize Latine-serving organizations to advocate collectively Increase Latine leadership and representation in policymaking Bring findings to state policymakers (e.g., NC General Assembly) Strengthen statewide partnerships and coalitions to share strategies and amplify impact Build sustainable, long-term funding for Latine health priorities
7. Improving access to trusted, culturally responsive providers and community organizations	Fragmented, uncoordinated service systems Difficulty navigating disconnected healthcare and social services Shortage of trusted, bilingual/culturally responsive providers Insufficient language justice beyond interpreters Lack of workforce pipeline for bilingual Latine providers	Build partnerships among healthcare, community, and faith-based organizations Strengthen cross-organizational communication and streamline referrals Map community assets (e.g., bilingual providers, community health workers, and cultural organizations) Improve interpretation services and hire bilingual Latine staff across roles Establish pathways and workshops to increase Latine health professionals Ensure consistent, linguistically appropriate information and communication across all service systems
8. Addressing social determinants of health	Chronic diseases (diabetes, obesity, and cardiovascular disease) linked to nutrition and economic instability Limited access to healthy and affordable food Housing instability and overcrowding Barriers to employment, living wages, and transportation Limited preventive care (e.g., screenings and vaccines) Stress related to immigration, discrimination, and economic pressures Disproportionate challenges in rural areas	Strengthen partnerships with public health, housing, workforce, and food security programs Expand affordable housing and food access initiatives Support culturally grounded nutrition and chronic disease education Promote policies supporting living wages and worker protections Improve transportation and childcare supports Integrate social needs screening and referrals into clinics and community programs

## Data Availability

The data is not publicly available due to privacy and ethical considerations. The dataset includes detailed perspectives from Spanish-speaking Latine community members, including immigrants; disclosure could increase the risk of participant identification and potential harms such as discrimination, stigma, or exposure of immigration status that may affect safety or willingness to seek services. The dataset also includes perspectives that could pose professional or personal risks if disclosed. Consistent with our community-engaged approach and agreements with partners, data are available from the corresponding author upon reasonable request and with appropriate safeguards in place.
